# Potential effects of probiotics on atherosclerosis

**DOI:** 10.20517/mrr.2024.22

**Published:** 2024-12-21

**Authors:** Gerrit A. Stuivenberg, Annabel Poon, Jeremy P. Burton, J. David Spence

**Affiliations:** ^1^Microbiology and Immunology, Western University, London N6A 3K7, Canada.; ^2^The Canadian Centre for Human Microbiome and Probiotic Research, Lawson Health Research Institute, London N6A 4V2, Canada.; ^3^Stroke Prevention and Atherosclerosis Research Centre, Robarts Research Institute, Western University, London N6G 2V4, Canada.

**Keywords:** Probiotics, atherosclerosis, uremic toxins, gut microbiota

## Abstract

The rising global incidence of atherosclerosis highlights the inadequacies in our understanding of the pathophysiology and treatment of the disease. Increasing evidence outlines the importance of the intestinal microbiome in atherosclerosis, wherein gut-derived uremic toxins (GDUTs) may be of concern. Plasma levels of the GDUTs trimethylamine n-oxide (TMAO), *p*-cresyl sulfate, and indoxyl sulfate are associated with accelerated renal function decline and increased cardiovascular risk. Thus, reducing the amount of GDUTs in circulation is expected to benefit patients with atherosclerosis. Because some beneficial bacteria can clear GDUTs *in vitro* and *in vivo*, orally administered probiotics targeting the intestinal tract represent a promising way to bring about these changes. Atherosclerosis such, this perspective reviews the potential use of probiotics to treat atherosclerosis, particularly in patients with non-traditional risk factors and/or impaired renal function.

## INTRODUCTION

Atherosclerosis is a chronic inflammatory disease characterized by the thickening of arteries due to plaque accumulation, and narrowing of arteries due to plaque rupture and thrombosis. Atherosclerosis progression is strongly dependent on dyslipidemia, hypertension, and inflammatory responses, and thus, traditional risk factors such as age, sex, diabetes, blood pressure, and smoking are thought to account for most cases. Many readers may be surprised that a probiotic could be leveraged to improve atherosclerosis outcomes. However, as shown in Table 1, traditional risk factors account for only a small proportion of atherosclerosis, and toxic metabolites formed by the gut microbiota emerged as strong predictors of atherosclerosis severity^[1]^, measured as carotid total plaque area (TPA)^[2]^.

**Table 1:** Backward linear regression of total plaque area (cube root transform)

**Model summary**						
**Model**		**R**		**R square**	**Adjusted R square**	**Std. error of the estimate**
12		0.517		0.267	0.245	2.66330
**Coefficients**						
**Model**		**Unstandardized coefficients**		**Standardized coefficients**	**t**	**Sig.**
		**B**	**Std. error**	**Beta**		
12	(Constant)	0.047	1.566		0.030	0.976
	Age	0.074	0.019	0.239	3.877	< 0.001
	Serum high-density lipoprotein	-1.169	0.511	-0.155	-2.288	0.023
	P-cresyl sulfate	0.017	0.006	0.179	2.809	0.005
	Pack-years smoking	0.041	0.009	0.280	4.569	< 0.001
	Serum triglycerides	-0.497	0.185	-0.183	-2.691	0.008
	TMAO	0.069	0.030	0.149	2.307	0.022
Dependent variable: cube root transform of TPA mm^2^.						
**Excluded variables**						
**Model**		**Beta in**	**t**	**Sig.**	**Partial correlation**	**Collinearity statistics**
						**Tolerance**
12	Total choline (mg)	-0.062	-1.003	0.317	-0.071	0.955
	Phenyl sulfate	-0.005	-0.085	0.933	-0.006	0.883
	Diabetes mellitus	-0.005	-0.081	0.936	-0.006	0.873
	Hippuric acid	0.027	0.425	0.672	0.030	0.899
	Indoxyl sulfate	-0.027	-0.295	0.768	-0.021	0.450
	Diastolic blood pressure	0.023	0.374	0.709	0.026	0.960
	P-cresyl glucuronide	-0.001	-0.016	0.988	-0.001	0.600
	Phenylacetylglutamine	0.048	0.545	0.587	0.038	0.463
	Total choline, no supplements (mg)	-0.063	-1.016	0.311	-0.071	0.951
	Systolic blood pressure	0.055	0.889	0.375	0.063	0.956
	Sex	-0.065	-0.977	0.330	-0.069	0.809

Reproduced by permission of Elsevier from^[1]^. TMAO: Trimethylamine n-oxide.

There is increasing evidence to support the role of gut microbiota in both the progression and prevention of atherosclerosis. Jie *et al.* showed that the gut microbiota of atherosclerosis patients distinguished them from healthy controls and could predict atherosclerosis risk^[3]^. Microbial metabolites produced in the gastrointestinal tract, such as trimethylamine N-oxide (TMAO)^[4]^ and the short-chain fatty acid (SCFA) propionate^[5]^, have been linked to atherosclerosis severity and protection, respectively. *Lactobacillus rhamnosus* is enriched in stable atherosclerotic plaques, suggesting it may have a role in regulating plaque stability^[6]^. Together, these findings highlight the ability of resident microbes to influence plaque growth and stability.

Orally delivered probiotics are an effective way of altering the metabolic flux of the gut microbiota to influence patient outcomes, because the distribution of metabolites produced by the network of gut microbes will change. For established risk factors of atherosclerosis, probiotics could help equilibrate lipid metabolism, vascular function, and macrophage polarization, which would benefit patients. Thus, probiotics have promise in managing the newly emerging risk factors of atherosclerosis by altering gut microbiota composition^[7,8]^ and clearing harmful gut-derived uremic toxins (GDUTs)^[9-11]^. The aim of this perspective is to assess whether probiotics may improve atherosclerosis, with a focus on emerging risk factors. We also provide recommendations on how to identify novel probiotic candidates.

## EXTREMES OF ATHEROSCLEROSIS HIGHLIGHT THE IMPORTANCE OF THE GUT MICROBIOTA

**Figure 1:**
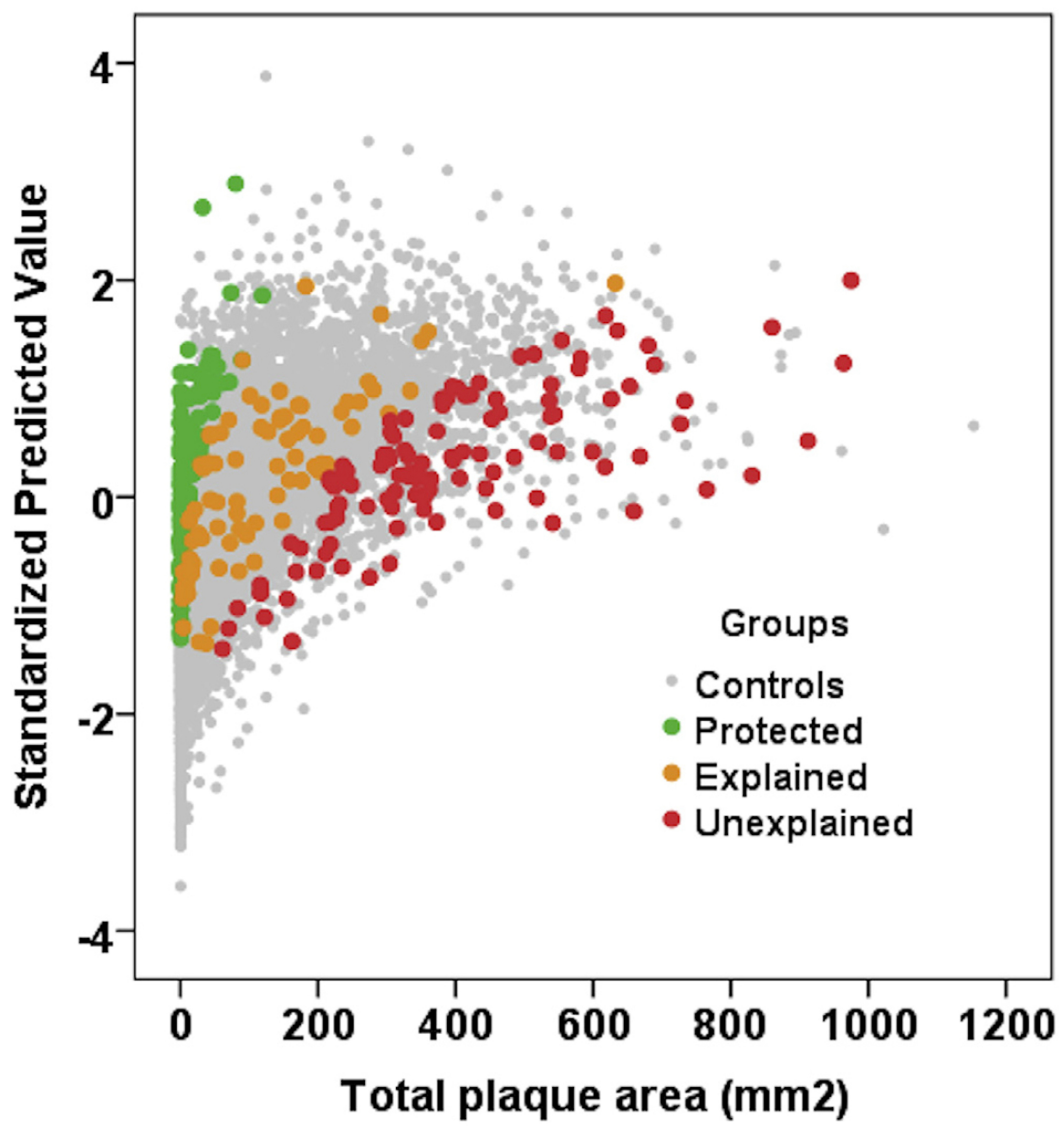
Distribution of the study participants among the clinic population of 3,056 patients in the linear regression model. Measured TPA is plotted against the standardized predicted value of plaque area based on the risk factors in the regression model: age, sex, diabetes, smoking status, serum creatinine, systolic and diastolic blood pressures, total cholesterol, triglycerides, LDL-C, and HDL-C. Control patients are shown as grey dots. The red dots represent study participants with residual scores > 2, i.e., they are the 5% extreme cases with unexplained atherosclerosis, with more plaque than predicted by risk factors by 2 standard deviations or more. Green dots represent protected patients; these are the 5% extremes with much less plaque than predicted, with residual scores < -2; orange dots represent patients with explained atherosclerosis, whose plaque burden is predicted by the risk factors, with residual scores between -2 and 2. Reproduced by permission of Elsevier from^[1]^. TPA: Total plaque area; LDL-C: low-density lipoprotein-C; HDL-C: high-density lipoprotein-cholesterol.

Carotid plaque burden is strongly associated with cardiovascular risk^[12,13]^. Among patients in whom TPA and levels of traditional risk factors have been measured, three phenotypes can be distinguished: Explained atherosclerosis, in which plaque burden is explained by traditional risk factors; Unexplained atherosclerosis, wherein atherosclerosis is severe despite little risk; and Protected individuals, who have high levels of traditional risk factors, but little or no carotid plaque. Figure 1 shows the distribution of these phenotypes among 316 of 3,056 patients who attended the Stroke Prevention & Atherosclerosis Research Centre, Robarts Research Institute, London, Canada.

In that study, metabolic products of the gut microbiota that typically accumulate in individuals with renal failure, GDUTs, were assayed in plasma by ultra-performance liquid chromatography coupled to quadrupole time-of-flight mass spectrometry. Plasma levels of TMAO, *p*-cresyl sulfate, *p*-cresyl glucuronide, and phenylacetylglutamine were significantly lower among patients with the protected phenotype, and higher in those with the unexplained phenotype, despite no significant differences in renal function or in dietary intake of nutrient precursors of GDUT. As shown in Table 1, both TMAO and *p*-cresyl sulfate were significant predictors of TPA in a model that excluded diabetes, sex and systolic blood pressure, i.e., these intestinal metabolites were stronger predictors of TPA than those three traditional risk factors. While our study did not show a significant effect on TPA by other GDUTs, such as indoxyl sulfate, some have been implicated to promote atherosclerosis, and will be discussed later in the review.

## USING PROBIOTICS TO REDUCE GUT MICROBIOTA METABOLITES RELEVANT TO ATHEROSCLEROSIS

The atherosclerosis-relevant probiotic studies conducted on humans and mice are summarized in Tables 2 and 3, respectively.

**Table 2:** Summary of human studies using probiotics relevant to atherosclerosis

**Article**	**Strain(s)**	**Summary/main findings**
The effect of yoghurt and its probiotics on blood pressure and serum lipid profile; a randomised controlled trial	● *Lactobacillus acidophilus* La5 ● *Bifidobacterium animalis* subsp. lactis Bb12	● 156 overweight men and women over 55 years old ○ 4 intervention groups: ■ Probiotic yogurt + probiotic capsules ■ Probiotic yogurt + placebo capsules ■ Control milk + probiotic capsules ■ Control milk + placebo capsules ■ Probiotic yogurt did not significantly change blood pressure, heart rate, or serum lipid concentrations ■ Probiotic capsules did not change blood pressure or concentrations of total cholesterol or triglycerides
*Lactobacillus plantarum* 299v supplementation improves vascular endothelial function and reduces inflammatory biomarkers in men with stable coronary artery disease	● Lp299v	● 20 men with stable coronary artery disease ○ Consumed drink containing Lp299v daily for 6 weeks ● Improved brachial flow-mediated dilation, endothelium-dependent vasodilation in resistance arteries ● Supplementation decreased circulating levels of IL-8, leptin ● The genus *Lactobacillus* was enriched in post-probiotic stool samples without other changes ● Lp299v improved vascular endothelial function and decreased systemic inflammation in men with coronary artery disease ○ Proposed circulating gut-derived metabolites responsible
Multispecies probiotic supplementation favorably affects vascular function and reduces arterial stiffness in obese postmenopausal women - a 12-week placebo-controlled and randomized clinical study	● *Bifidobacterium bifidum* W23 ● *Bifidobacterium lactis* W51 ● *Bifidobacterium lactis* W52 ● *Lactobacillus acidophilus* W37 ● *Lactobacillus brevis* W63 ● *Lactobacillus casei* W56 ● *Lactobacillus salivarius* W24 ● *Lactococcus lactis* W19 ● *Lactococcus lactis* W58	● 81 obese postmenopausal women aged 45-80 years ○ 3 interventions: ■ Placebo ■ Low probiotic dose ■ Original probiotic dose ● High dose intervention decreased: ○ Systolic blood pressure ○ Vascular endothelial growth factor ○ Pulse wave analysis systolic pressure, augmentation index and velocity ○ IL-6, TNFa, and thrombomodulin ● Low dose intervention decreased: ○ Systolic blood pressure ○ IL-6 ● Supplementation favorably modifies functional and biochemical markers of vascular dysfunction in obese postmenopausal women
Short Communication: effect of supplementation with *Lactobacillus casei* shirota on insulin sensitivity, β-cell function, and markers of endothelial function and inflammation in subjects with metabolic syndrome - a pilot study	● LcS	● 30 subjects supplemented with LcS for 12 weeks ● sVCAM-1 levels reduced in serum ● Did not improve parameters of insulin sensitivity, β-cell function, endothelial function, low-grade inflammation
Synbiotics easing renal failure by improving gut microbiology (SYNERGY) - a randomized trial	● Not specified (prebiotics and probiotics combined)	● Stage III and IV CKD patients ● Synbiotics administered for 6 weeks ● Reduced serum PCS but not IS ● Enriched *Bifidobacterium* in gut microbiota ● Increased albuminuria in the synbiotic arm
The influence of prebiotic arabinoxylan oligosaccharides on microbiota derived uremic retention solutes in patients with chronic kidney disease: a randomized controlled trial	● Arabinoxylan oligosaccharides	● Patients with stage III and IV CKD ● 4-week intervention with washout ● No significant changes in serum IS, PCS, or insulin resistance ● Slight reduction in TMAO levels
Probiotics-supplemented low-protein diet for microbiota modulation in patients with advanced chronic kidney disease (ProLowCKD): results from a placebo-controlled randomized trial)	● *Bifidobacterium longum* ● *Lactobacillus reuteri*	● Patients with stage III and IV CKD ● 3-month supplementation following a low-protein diet ● Probiotic group showed trends in reduced uremic toxins and proatherogenic markers ● Potential reduction in antihypertensive/diuretic use
Effects of probiotic supplementation on inflammatory biomarkers and uremic toxins in non-dialysis chronic kidney patients: a double-blind, randomized, placebo-controlled trial	● *Streptococcus thermophilus* ● *Lactobacillus acidophilus* ● *Bifidobacterium longum*	● Stage III and IV CKD patients ● Daily probiotics for 3 months ● Increased IL-6 levels
Randomized controlled trial of strain-specific probiotic formulation (Renadyl) in dialysis patients	● *Lactobacillus, Streptococcus* ● *Bifidobacterium species*	● Patients undergoing maintenance hemodialysis ● Two 2-month treatment periods with washout ● No significant efficacy confirmed due to the small sample size
Effect of short-term synbiotic treatment on plasma *p*-cresol levels in patients with chronic renal failure: a randomized clinical trial	● Not specified (synbiotic formulation)	● Patients with CKD stages 3-4 ● Synbiotic or placebo for 4 weeks ● Synbiotic lowered plasma *p*-cresol but did not affect gastrointestinal symptoms
An innovative synbiotic formulation decreases free serum indoxyl sulfate, small intestine permeability and ameliorates gastrointestinal symptoms in a randomized pilot trial in stage IIIb-IV CKD patients	● *Lactobacillus plantarum* ● *Lactobacillus acidophilus* ● *Bifidobacterium longum* ● *Bifidobacterium lactis* ● *Streptococcus thermophilus*	● CKD stage IIIb-IV patients ● Synbiotics administered for 2 months ● Reduced serum indoxyl sulfate and intestinal permeability ● Improved gastrointestinal symptoms
Effects of probiotics, prebiotics, and synbiotics on uremic toxins, inflammation, and oxidative stress in hemodialysis patients: a systematic review and meta-analysis of randomized controlled trials	● Various strains (review/meta-analysis of RCTs)	● Systematic review and meta-analysis of 23 RCTs on probiotics, prebiotics, and synbiotics in hemodialysis patients ● Decreased PCS, endotoxins, and inflammatory markers ● Improved antioxidant capacity
A randomized double-blind placebo-controlled trial of probiotics in post-surgical colorectal cancer	● *Lactobacillus acidophilus* BCMC 12,130 ● *Lactobacillus lactis* BCMC 12,451 ● *Lactobacillus casei* BCMC 12,313 ● *Bifidobacterium longum* BCMC 02120 ● *Bifidobacterium bifidum* BCMC 02290 ● *Bifidobacterium infantis* BCMC 02129	● 52 post-surgical colorectal cancer patients ● Randomized to receive placebo or probiotics for 6 months ● Reduced pro-inflammatory cytokines (TNF-α, IL-6, IL-10, IL-12, IL-17A, IL-17C, IL-22) in the probiotic group ● No significant effect on IFN-γ
Vitamin D and probiotic co-supplementation affects mental health, hormonal, inflammatory and oxidative stress parameters in women with polycystic ovary syndrome	● *Lactobacillus acidophilus* ● *Bifidobacterium bifidum* ● *Lactobacillus reuteri* ● *Lactobacillus fermentum*	● 60 women with polycystic ovary syndrome ● Randomized to receive Vitamin D + probiotics or placebo for 12 weeks ● Significant improvements in mental health (BDI, GHQ, DASS) ● Reduction in total testosterone, hirsutism, hs-CRP, MDA ● Improvement in TAC and GSH levels in the treatment group

Lp299v: *Lactobacillus plantarum* 299v; LcS: *Lactobacillus casei* Shirota; CKD: chronic kidney disease; PCS: *p*-cresyl sulfate; IS: indoxyl sulfate; TMAO: trimethylamine n-oxide; RCTs: randomized controlled trials; BCMC: registered probiotic trademark name from B-Crobes Laboratories Sdn. Bhd., Malaysia; BDI: beck depression inventory; GHQ-28: general health questionnaire-28; DASS: depression anxiety and stress scale; CRP: C-reactive protein; MDA: malondialdehyde; TAC: antioxidant capacity; GSH: glutathione.

**Table 3:** Summary of rodent studies using probiotics relevant to atherosclerosis

**Article**	**Strain(s)**	**Summary/main findings**
The anti-cholesterolaemic effect of a consortium of probiotics: an acute study in C57BL/6J mice	● *Lactobacillus acidophilus* CUL21, CUL60 ● *Bifidobacterium bifidum* CUL20 ● *Bifidobacterium animalis* CUL34 ● *Lactobacillus plantarum* CUL66	● C57BL/6J mice ● High-fat diet + probiotics for 2 weeks ● Reduced plasma cholesterol ● Increased fecal bile acids ● No effect on LDL/HDL ratio
Preventive effect and molecular mechanism of *Lactobacillus rhamnosus* JL1 on food-borne obesity in mice	● *Lactobacillus rhamnosus* JL1	● C57BL/6J mice ● High-fat diet + probiotics for 10 weeks ● Lower body weight ● Reduced liver index ● Improved lipid metabolism via the AMPK pathway
*Lactobacillus acidophilus* ATCC 4356 prevents atherosclerosis via inhibition of intestinal cholesterol absorption in apolipoprotein E-knockout mice	● *Lactobacillus acidophilus* ATCC 4356	● ApoE-/- mice ● Western diet + probiotics for 16 weeks ● Lower plasma cholesterol ● Reduced intestinal cholesterol absorption and atherosclerosis
The Lab4P consortium of probiotics attenuates atherosclerosis in LDL receptor deficient mice fed a high fat diet and causes plaque stabilization by inhibiting inflammation and several pro-atherogenic processes	● *Lactobacillus acidophilus* CUL21, CUL60 ● *Bifidobacterium bifidum* CUL20 ● *Bifidobacterium animalis* CUL34 ● *Lactobacillus plantarum* CUL66	● LDL receptor-deficient mice ● High-fat diet + probiotics for 12 weeks ● Reduced plaque burden ● Increased HDL ● Decreased LDL and plaque stabilization
*Lactobacillus mucosae* and *Bifidobacterium longum* synergistically alleviate immobilization stress-induced anxiety/depression in mice by suppressing gut dysbiosis	● *Lactobacillus mucosae* NK41 ● *Bifidobacterium longum* NK46	● C57BL/6 mice ● Immobilization stress + probiotics for 3 weeks ● Alleviated anxiety-like behaviors ● Reduced gut dysbiosis and inflammation
Probiotics alleviate oxidative stress in H_2_O_2_-exposed hepatocytes and t-BHP-induced C57BL/6 mice	● *Lactococcus lactis* MG5125 ● *Bifidobacterium bifidum* MG731 ● *Bifidobacterium animalis* MG741	● C57BL/6 mice ● Oxidative stress induced by t-BHP + probiotics ● Improved oxidative stress markers and hepatic enzyme activity
Antioxidant activity of *Lactobacillus plantarum* NJAU-01 in an animal model of aging	● *Lactobacillus plantarum* NJAU-01	● Kun Ming mice ● D-galactose-induced aging + probiotics ● Increased antioxidant enzymatic activities ● Reduced lipid oxidation
The probiotic Lactobacillus coryniformis CECT5711 reduces the vascular pro-oxidant and pro-inflammatory status in obese mice	● *Lactobacillus coryniformis* CECT5711	● C57BL/6J ● High-fat diet + probiotics for 12 weeks ● Improved endothelial function ● Reduced oxidative stress and inflammation

LDL: Low-density lipoprotein; HDL: high-density lipoprotein; AMPK: AMP-activated protein kinase.

### TMAO

**Figure 2:**
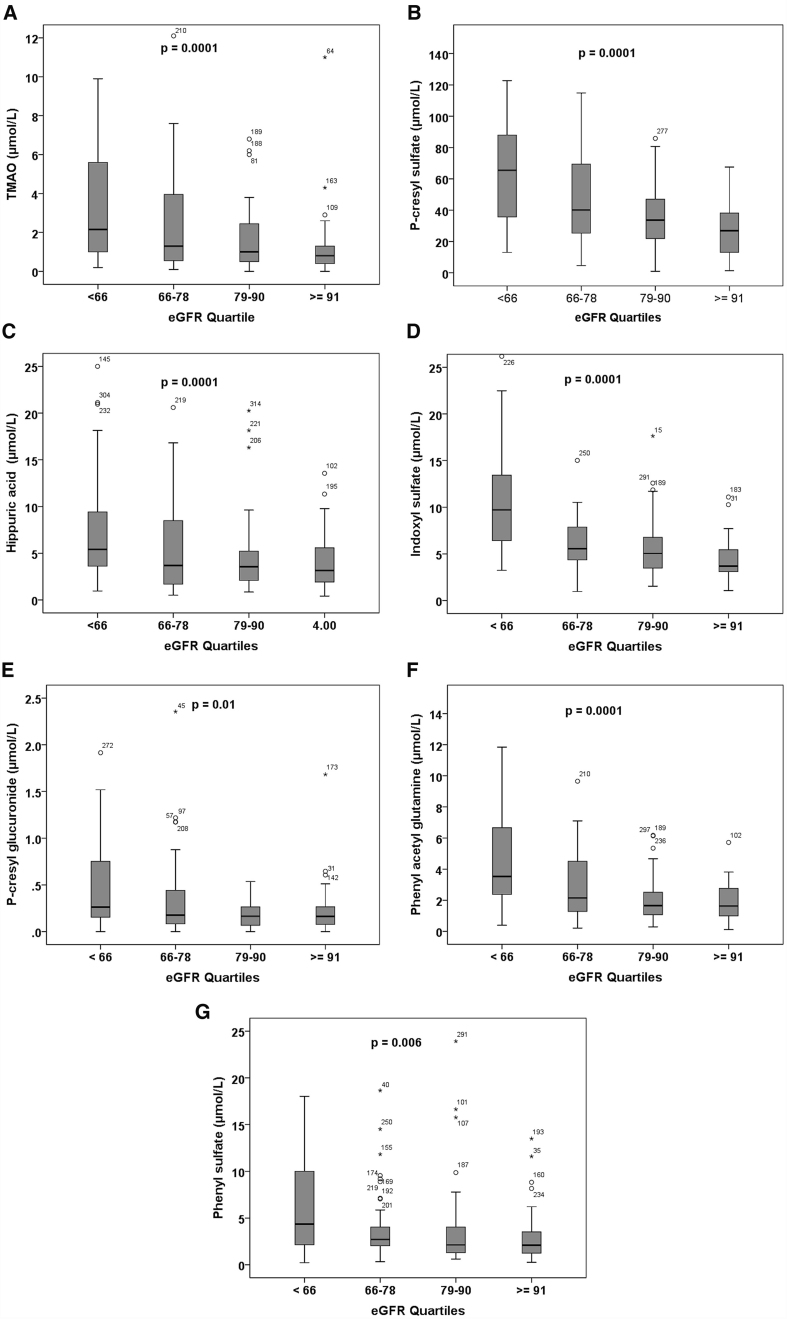
Plasma levels of GDUT by quartile of eGFR. Boxplots show plasma levels of GDUT in mmol/L by quartile of eGFR (calculated using the CKD-EPI equations). eGFR quartiles: Q1, ,66 mL/min per 1.73 m^2^; Q2, 66-78; Q3, 79-90; Q4, $ 91. (A) TMAO; (B) P-cresyl sulfate; (C) hippuric acid; (D) indoxyl sulfate; (E) P-cresyl glucuronide; (F) phenylacetyl glutamine; (G) phenyl sulfate. Plasma levels of all the metabolites were signiﬁcantly higher in lower quartiles of eGFR; *P* values were computed for differences in median values by the Kruskal-Wallis test because the distribution of plasma levels was not normally distributed. Reproduced by permission of Elsevier from^[20]^. GDUT: Gut-derived uremic toxin; eGFR: estimated glomerular filtration rate; CKD: chronic kidney disease; TMAO: trimethylamine N-oxide.

In the context of atherosclerosis, perhaps the best-known intestinal metabolite is TMAO. Phosphatidylcholine^[14]^ and carnitine^[15]^, which have high concentrations in animal-based protein sources, are converted to trimethylamine by gut bacteria, and then oxidized in the liver to TMAO^[16]^. Among patients referred for coronary angiography, TMAO levels in the top quartile were associated with a 2.5-fold increase in the risk of myocardial infarction, stroke, or vascular death over 3 years^[17]^. The harmful effects of TMAO include thrombogenesis^[18]^, accelerated renal function decline, and increased mortality^[19]^. Figure 2 shows that levels of TMAO and other intestinal metabolites are increased with even moderate impairment of renal function^[20]^, reaching levels that are normal in older patients. As such, elevated TMAO explains, in part, the elevated risk of developing atherosclerosis in patients with impaired renal function^[21]^.

Indeed, dietary and pharmacological treatments to lower TMAO have been developed; for instance, switching from red meat to white meat or vegetarian/vegan meals significantly reduced plasma TMAO over one month^[22]^. Additionally, two inhibitors are currently under development: one targets choline trimethylamine lyase^[23]^, a bacterial enzyme involved in generating trimethylamine from nutrients such as phosphatidylcholine and carnitine, while the other inhibits flavin-containing monooxygenase 3^[24]^, a hepatic enzyme that converts trimethylamine into TMAO. Given the microbial origins of the metabolite, the potential for probiotics to reduce plasma TMAO is also a subject of inquiry.

It is well established that the gut microbiota composition of atherosclerosis patients drastically differs from that of healthy controls. Typically, these patients present an increased abundance of *Enterobacteriaceae* and *Streptococcus* species^[3]^. However, additional studies have also linked subclinical markers of atherosclerosis to increased levels of *Collinsella*, where the healthy controls had more *Roseburia* and *Eubacterium*^[25]^. In addition, bacterial nucleic acids originating from the genera *Chryseomonas*, *Veillonella*, and *Streptococcus* were detected in atherosclerosis plaques, with several of these phylotypes also being identified in the gut^[25,26]^. Furthermore, a metagenome-wide association study revealed that atherosclerosis patients differ from healthy controls not only in gut microbiome composition, but also in its functional and metabolic capacity^[3]^. This is particularly true for the metabolism and transport of molecules crucial for cardiovascular health, such as TMAO, taurine, and sphingolipids^[3,25]^. Ultimately, these microbial and functional alterations may contribute to the development and progression of atherosclerosis through various mechanisms^[3,25,26]^, which are described in greater detail throughout this perspective. Considering that one method of action of probiotics is to increase the relative abundance of certain beneficial microbes to shift the microbiome toward a composition more similar to that of healthy control^[27]^, it is not surprising that probiotics are being considered therapeutic for atherosclerosis.

Strains of lactobacilli and bifidobacteria are common in probiotic formulations and tend to be the first bacteria investigated for new microbial therapies, including those for atherosclerosis. Treatment with oral *Lactobacillus acidophilus* NCFM or *L. acidophilus* BG2F04 decreased dimethylamines, potential precursors for trimethylamine production^[28]^. There is also evidence that some lactobacilli can directly mitigate the harmful effects of TMAO directly as it relates to atherosclerosis. In mice, the oral supplementation of *Lactiplantibacillus plantarum* CGMCC 8198 slowed atherosclerosis progression by reducing inflammation and oxidative stress caused by TMAO. There is also evidence to support *Lactocasebacillus rhamnosus* GG as an efficient TMAO-reducing strain in both humans and animal models^[29]^. Interestingly, the effect of *L. rhamnosus* may extend beyond the gut. While present in both stable and unstable (or actively growing) atherosclerotic plaques, *L. rhamnosus* DNA had greater enrichment in stabilized plaques^[6]^. Indeed, the details of how nucleic acid components of probiotics are enriched in plaques and how they may slow atherosclerosis progression are not clear. Certainly, the DNA of other bacterial species is present in these plaques, and thus, stability may rely on community dynamics as opposed to a single species or strain. *Enterobacter aerogenes* ZDY01 was also inquired for its probiotic potential and was shown to attenuate choline-induced atherosclerosis in mice by decreasing cecal TMA and promoting reverse cholesterol transport^[30]^. While these results highlight the real possibility that probiotics could reduce TMAO burden, most data come from animal studies or small-cohort clinical trials. Further testing of these probiotics in larger populations of human atherosclerosis patients is required to establish efficacy and the potential for any associated risks.

### Indoxyl sulfate and *p*-cresyl sulfate

As shown in Table 1, *p*-cresyl sulfate was a stronger predictor of TPA than TMAO. It would be desirable, therefore, to develop therapies to reduce not only the plasma levels of TMAO, but also *p*-cresyl sulfate and other toxic GDUTs, such as indoxyl sulfate, which have been shown to promote atherosclerosis. Indoxyl sulfate and *p*-cresyl sulfate are produced by the gut microbiota through the fermentation of tryptophan and tyrosine, respectively. These toxins are commonly associated with chronic kidney disease, where their accumulation occurs due to impaired renal function^[31-33]^. However, indoxyl sulfate and *p*-cresyl sulfate are also closely linked to cardiovascular diseases and mortality^[34-36]^. In atherosclerosis patients, these toxins are elevated in the plasma, but this observation cannot be explained by kidney function or the intake of dietary precursors^[1]^. This suggests the gut microbiota of atherosclerosis patients has a heightened ability to produce GDUTs, leaving them at risk of the deleterious effects of these toxins. Furthermore, there are very few strategies available for their elimination, and most of these strategies impart an incomplete effect^[37]^.

To date, bifidobacteria have demonstrated the greatest success in preventing the build-up of *p*-cresyl sulfate and its microbial precursor, *p*-cresol. Probiotic formulations containing at least one strain of *Bifidobacterium longum* have been shown to reduce plasma *p*-cresol in two independent studies after 3 months of supplementation^[38,39]^. Another study that used a microbial mixture of *Bifidobacterium infantis*, *Lactobacillus acidophilus*, and *Enterococcus faecalis* showed oral supplementation of these microbes reduced fecal and serum *p*-cresol. A synbiotic therapy (probiotic + prebiotic) containing bifidobacteria was also shown to significantly reduce *p*-cresyl sulfate in a placebo-controlled trial^[40]^. These findings indicate the strong potential to use probiotics as a treatment to reduce GDUT. However, due to the existence of a handful of studies reporting probiotic mixtures did not reduce levels of *p*-cresol or *p*-cresyl sulfate, the efficacy of probiotics for this purpose remains unclear^[41-44]^. It is of interest to note, however, that the trials where no decrease in GDUTs was observed utilized multi-strain probiotics and not an individual bacterium^[40-45]^. Delivering bacteria in different combinations alters their activity and potentially blocks their beneficial functions. Highlighting this point, the oral delivery of five different combinations of beneficial bacteria to nephrectomized rats showed that only two could reduce blood urea nitrogen and serum creatinine despite the similar consortia^[46]^. While the use of multi-strain probiotics certainly has merit in some cases, care should be taken to ensure that the beneficial properties are maintained when delivered alongside other strains.

When considering individual strains, both *Bifidobacterium breve* and *Bifidobacterium longum* can reduce toxin burden and enhance SCFA production^[47-51]^, both of which would be beneficial to atherosclerosis patients. While the mechanism of toxin clearance is often unknown, both *B. breve* and *B. longum* have been shown to clear *p*-cresol directly from bacteriological growth media^[10]^ and a colonic environment^[52,53]^. Taken together, these studies suggest that oral supplementation of *B. breve* or *B. longum* could offer protection against gut microbiota metabolites relevant to atherosclerosis. These studies also point toward the importance of proper strain selection. In the future, there should be an emphasis on testing individual probiotic strains for the desired function, whether toxin clearance or any other, and ensuring that these activities are retained when the strains are delivered in combination.

There have been fewer studies focusing on the clearance of indoxyl sulfate using probiotics; however, they are still worth considering. In cohorts of hemodialysis patients, who often have a high burden of uremic toxins, three studies assessed probiotics using different strains of *B. longum*. Two of these studies showed a clear decrease in indoxyl sulfate^[48,50]^ after 5 weeks of oral administration, while another indicated decreased indoxyl glucuronide without reaching traditional measures of significance^[41]^. Beyond probiotics, AST-120, an oral activated carbon supplement, reduces the serum and urine levels of indoxyl sulfate in patients with uremia by adsorbing indole in the intestines, thereby increasing its excretion into feces^[54]^. Unfortunately, AST-120 often requires the patient to consume 30 or more capsules daily, not considering any other medications they may be taking^[54,55]^. It would, therefore, be beneficial to identify probiotic strains that could effectively reduce indole in the gut, similar to what has been done for *p*-cresol, the microbial precursor of *p*-cresyl sulfate.

## PROBIOTICS FOR TRADITIONAL RISK FACTORS OF ATHEROSCLEROSIS

Beyond reducing metabolites produced by the intestinal microbiota, probiotics have also shown great potential in protecting against traditional atherosclerosis risk factors such as lipid metabolism, endothelial dysfunction, and inflammation.

### Cholesterol

Maintaining a well-controlled cholesterol equilibrium is paramount in slowing atherosclerosis progression^[56-59]^. Regrettably, numerous inquiries into antiatherosclerotic drugs have yielded disappointing results^[60-62]^. Alternatively, there have been various reports of probiotics having favorable impacts on lipid metabolism, suggesting their potential utility in atherosclerosis patients.

Several strains of lactobacilli have demonstrated robust hypocholesterolemic effects *in vivo*^[56-59]^. Among these, the Lab4 probiotic consortium with *Lactobacillus plantarum* CUL66 stood out because it caused a notable reduction in total plasma cholesterol levels while also mitigating diet-induced weight gain^[63]^. It must be noted that these studies were conducted in mice, necessitating further validation in human cohorts before any assertions can be made regarding their efficacy against atherosclerosis. The use of probiotics to reduce serum cholesterol remains blurred. For instance, a clinical trial utilizing *Lactobacillus acidophilus* and *Bifidobacterium animalis* via yogurt or capsules did not improve atherosclerosis risk factors in overweight individuals^[64]^. The lack of success in this study does not negate the potential effectiveness of probiotics. Researchers often rely on commercially available strains lacking documented activity against the disease. Therefore, future clinical trials should focus on utilizing strains with established cholesterol-lowering properties, such as those identified in the murine investigations^[63,65-67]^.

### Inflammation

The orchestration of the inflammatory response is a pivotal determinant of atherosclerosis progression and stability^[68]^. Within this complex milieu, cytokines, notably TNF-α, CRP, and IL-6, emerge as influential players in atherosclerosis development. During the nascent stages of atherosclerosis, these cytokines act as catalysts, inciting endothelial cell activation, amplifying the synthesis of adhesion molecules, and leading to the migration of immune cells^[69]^. As atherosclerosis advances to later stages, the presence of proinflammatory cytokines manifests as plaque rupture and thrombosis. Thus, promoting an anti-inflammatory environment raises hope for atherosclerosis treatment, which has been corroborated by compelling clinical trials^[70,71]^.

The production of TNF-α within atherosclerotic plaques and elevated levels of the compound in the bloodstream correlate with atherosclerosis progression^[72]^. Recent investigations have highlighted a consortium of probiotics such as *Lactobacillus mucosae* NK41, *Bifidobacterium longum* NK46, *Bifidobacterium breve* DSM 16604 and DSM 24706 that can reduce TNF-α *in vivo*^[73,74]^. While the mechanism behind this observation is still unclear, this nuanced influence of probiotics on the release of inflammatory mediators appears to be intertwined with altered miRNA expression within the gastrointestinal tract^[75]^. For example, a strain of *Lactobacillus plantarum* has been shown to reduce TNF-α mRNA expression over the span of three weeks, likely by regulating miRNA-450a expression^[76]^. In contrast, a clinical investigation of the same strain showed that there were no substantial shifts in TNF-α, IL-6, IL-1b, nor cortisol after a four-week period, compared to placebo^[77]^. The difference in outcomes underscores the need to tailor probiotic interventions to the unique attributes of study participants, because the second intervention was in patients with depression, not atherosclerosis. Elevated IL-6 has also emerged as a biomarker for atherosclerosis risk and progression^[78,79]^. Some probiotics have been shown to reduce the presence of such inflammatory cytokines. Indeed, a study involving colon cancer patients revealed that the postoperative administration of a probiotic blend containing multiple strains of lactobacilli and bifidobacteria for four weeks resulted in a marked reduction in proinflammatory cytokines TNF-α, IL-6, and IL-22 in peripheral blood^[80]^. These findings and others from animal studies are promising, but there is a need for randomized, double-blind human trials to authenticate the anti-inflammatory potential of probiotics in atherosclerosis patients.

C-reactive protein (CRP) is another inflammatory marker produced in response to cytokines like IL-6, IL-1β, and TNF-α, and is associated with vascular endothelial cell impairment, thrombosis, and ultimately atherosclerosis progression^[81,82]^. Detectable within early lesions, CRP levels also increase as atherosclerosis advances to later stages^[83]^. To date, some studies investigating the impact of probiotics on CRP levels have been successful. An 8-week course of probiotic capsules containing *L. acidophilus*, *Lactobacillus casei*, and *Bifidobacterium bifidum* significantly reduced CRP^[84]^. Another study involving a probiotic capsule containing four strains, combined with vitamin D administration for 12 weeks, demonstrated decreased CRP in women with polycystic ovarian syndrome^[85]^. While direct evidence linking gut microbiota modulation of inflammatory factors to atherosclerosis progression remains inconclusive, these studies offer valuable insights and avenues for exploring novel preventive and therapeutic strategies for atherosclerosis.

While further research is needed to validate the efficacy of probiotics in modulating inflammatory factors and slowing atherosclerosis advancement, these findings pave the way for innovative strategies in preventing and treating atherosclerosis.

### Endothelial function

Endothelial dysfunction is a key event in the early stages of atherosclerosis development/progression and is caused by disrupted vascular homeostasis due to oxidative stress and inflammation^[86,87]^. Probiotics can reduce oxidative stress, quell vascular inflammation, and increase nitric oxide (NO) production, each of which could independently delay the progression of atherosclerosis^[88-90]^.

Oxidative stress promotes endothelial dysfunction by impairing the vascular endothelium, causing inflammation, and oxidizing low-density lipoprotein-C (LDL-C)^[91-93]^. Restoring the endothelial structure could improve atherosclerosis outcomes, and probiotics have shown some promise in this area. For instance, probiotic Kefir, a fermented beverage that can be prepared using either milk or water and kefir grains that are combined with a starter culture that contains a combination of yeast species and lactic acid bacteria, has been associated with enhanced endothelial function and vascular structure repair in animal studies^[94]^. Furthermore, specific probiotics such as *Lactococcus lactis* MG5125, *Bifidobacterium bifidum* MG731, and *Bifidobacterium animalis* MG741 have showcased antioxidant properties by bolstering total antioxidant capacity and mitigating oxidative stress^[95-98]^. *Lactobacillus plantarum* NJAU-01 and *Lactobacillus fermentum* DR9 have demonstrated the ability to decrease lipid oxidation levels and enhance antioxidant enzyme activity in rats^[99,100]^. These discoveries indicate that probiotics have potential as therapeutic interventions to prevent diseases associated with oxidative stress like atherosclerosis but require further testing in human cohorts.

Reduced production and sensitivity of NO can also disrupt vascular equilibrium^[101-103]^. Using probiotics to enhance NO production and availability may be a new approach to combat atherosclerosis. For instance, *Lactobacillus coryniformis* CECT5711 has been shown to inhibit certain inflammatory markers in obese mice and improve endothelial function by increasing NO levels^[104]^. Similarly, *Lactobacillus casei* boosts NO production in some human cells^[105]^. Nevertheless, the impact of probiotics on NO production in vascular endothelial cells or animal models has not been tested. Considering that endothelial cells are a primary source of NO via NO synthase expression, how probiotics regulate NO production in these cells should also be inquired^[106]^. Furthermore, *Lactobacillus fermentum* CECT5716 has been reported to counteract the endothelial dysfunction induced by certain medications by reducing oxidative stress and inflammation in the blood vessels^[107]^. These findings suggest that probiotics may help alleviate vascular manifestations by targeting and suppressing pathways related to oxidative stress.

While all the strains mentioned in this section so far have only been shown to have activity in animal or cell-based models, clinical studies have also reported that certain probiotics can improve endothelial dysfunction. In a 12-week trial involving 81 participants, a probiotic that contained *Lactobacillus*, *Bifidobacterium*, and *Lactococcus* reduced systolic blood pressure, inflammatory markers, and parameters linked to endothelial dysfunction^[108]^. However, not all probiotics exhibit these clinical benefits. For example, *Lactobacillus casei* Shirota (LcS) did not show significant improvements in low-grade inflammation or endothelial dysfunction in patients with metabolic syndrome^[109]^. These studies highlight the need for further research in atherosclerosis patients and with larger sample sizes to better understand the impact of probiotics on endothelial function.

## HOW TO FIND THE RIGHT PROBIOTIC FOR ATHEROSCLEROSIS?

In general, selecting a probiotic strain for a target disease should be based on recommendations from in-depth reviews or organizations such as the Food and Drug Administration or the European Society for Paediatric Gastroenterology, Hepatology and Nutrition. In our opinion, the available pool of research seems insufficient to provide an accurate recommendation for an atherosclerosis-specific probiotic. At present, there are no probiotic recommendations for atherosclerosis within the Canadian or American websites of clinically documented probiotic strains and products (www.usprobioticguide.com, accessed on 28 February 2024; www.probioticchart.ca, accessed on 28 February 2024). A major concern we have is that most of the studies mentioned in this perspective measured the effect of probiotics on factors relevant to atherosclerosis, such as inflammation, but were not performed in atherosclerosis patients. Therefore, while these strains show promise in managing some of the factors associated with atherosclerosis, they must be tested in an appropriate cohort. As such, our current suggestions will focus on how to design new studies to identify and assess useful probiotic strains to treat atherosclerosis.

### Finding new strains

In an ideal situation, performing *in vitro* experiments before any human or animal studies would assist in identifying beneficial strains with specific target activities related to atherosclerosis. However, as we have mentioned, studies often rely on commercially available strains rather than those recognized for their specific traits.

We believe that the identification of strains relevant to atherosclerosis, or any other disease should follow a series of simple steps. First, strains should be sequenced to identify useful genes harbored within their genetic content. The utility of these genes should then be proven by demonstrating the activity *in vitro* (e.g., reduction in uremic toxins or serum cholesterol). Strains with sufficient activity can then be assessed using relevant cell culture and animal models. The final step would then be to perform a well-designed clinical trial in an appropriate population of humans (i.e., individuals with a certain disease or condition). Certainly, this concise set of steps represents a more appropriate selection process than choosing bacterial strains based solely on availability. However, it is crucial to highlight that this method of strain selection overlooks the essential need to assess each strain for stability and safety before introducing it to a group of at-risk individuals (i.e., atherosclerosis patients). The discussion on how to conduct these evaluations on probiotic strains is omitted here as it has been comprehensively addressed elsewhere^[110-113]^.

## HOW SHOULD STRAINS BE ASSESSED CLINICALLY?

When evaluating strains in clinical settings, researchers should utilize randomized, double-blind placebo-controlled studies to reduce bias. Trials should ideally be carried out in target populations, in this case, atherosclerosis patients, alongside standard treatments to avoid interfering with patients’ medical care. To date, only a handful of clinical trials have investigated how probiotics directly influence atherosclerosis patients, with most of the research being conducted in mouse or rat models. While these studies demonstrate the feasibility of the approach, they do not provide concrete evidence to support the widespread adoption of a specific strain or product.

After identifying beneficial strains with proven clinical efficacy against atherosclerosis, the challenge of determining how to assess the results of various strains emerges. Should the focus be on probiotics that alleviate inflammation or promote cholesterol clearance? Is a combination of strains more effective than one alone? The answer is not straightforward, and in fact, the existence of a “one-size-fits-all” probiotic for atherosclerosis is unlikely. Individuals respond differently to the physiological challenges of atherosclerosis, as demonstrated by the existence of a protected phenotype [Figure 1], and thus, patients may also have similar responses to treatment wherein one patient might benefit more from a cholesterol-reducing probiotic while another might respond better to that with anti-inflammatory action. By assembling a diverse range of strains with various activities against atherosclerosis symptoms and risk factors, this potential issue could be overcome.

## CONCLUSIONS

This review focused on highlighting the potential for probiotics to protect against both the traditional and non-traditional risk factors of atherosclerosis. Probiotics show promise as therapies for atherosclerosis, with the possibility of plaque stabilization and a reduced risk of cardiovascular events. The microbial supplements mainly consisted of lactobacilli or bifidobacteria and were often tested using animal models. The limited number of quality clinical trials in atherosclerosis patients and conflicting findings make strain recommendation for atherosclerosis difficult. Therefore, more studies are needed to identify new strains with specific metabolic functions against atherosclerosis and to test them in the appropriate human populations. Long-term studies that also address how probiotic administration improves not only symptoms and quality of life but also additional clinical parameters such as plaque burden, cardiovascular events, and survival are needed.

## DECLARATIONS

### Copyright

© The Author(s) 2024.
